# Quantum Transport Through Tunable Molecular Diodes

**DOI:** 10.1038/s41598-017-07733-4

**Published:** 2017-08-04

**Authors:** Joshua Tobechukwu Obodo, Altynbek Murat, Udo Schwingenschlögl

**Affiliations:** King Abdullah University of Science and Technology (KAUST), Physical Science and Engineering Division (PSE), Thuwal, 23955-6900 Saudi Arabia

## Abstract

Employing self-interaction corrected density functional theory combined with the non-equilibrium Green’s function method, we study the quantum transport through molecules with different numbers of phenyl (donor) and pyrimidinyl (acceptor) rings in order to evaluate the effects of the molecular composition on the transport properties. Excellent agreement with the results of recent experiments addressing the rectification behavior of molecular junctions is obtained, which demonstrates the potential of quantum transport simulations for designing high performance junctions by tuning the molecular specifications.

## Introduction

Organic electronics is expected to play a key role in future semiconductor industry, as it costs typically less to process an organic than an inorganic semiconductor^1^. Furthermore, promising intrinsic device functionalities are available, such as the rectification in a molecular junction^2–5^. Being determined by the ability of a system to support current differently in foward and backward direction, molecular diodes were early analyzed by Aviram and Ratner^6^. They exploit the energy gap between the highest occupied molecular orbital (HOMO) of the donor and the lowest unoccupied molecular orbital (LUMO) of the acceptor. Various similar concepts later have been proposed theoretically and demonstrated experimentally^7–12^. In a single molecule rectifier typically a donor-acceptor molecule is placed between Au electrodes using a thiol group as linker because of its high flexibility under stress^13^. For example, such contacts can be achieved by mechanical break junction techniques^14, 15^. The effects of solvents on molecular junctions and their rectification properties recently have been studied in refs 16, 17. From a fundamental perspective, however, the rectification behavior is not yet well understood, despite important advances^18^.

Recent experiments have studied the charge transport through tetraphenyl and dipyrimidinyl-diphenyl molecular diodes^9, 19, 20^, finding a pronounced rectification in the latter case, with a larger current in the direction from dipyrimidinyl (acceptor) to diphenyl (donor). Since these observations suggest that the properties of the junction can be tuned by chemical substitution, we study the rectification behavior of a series of molecular diodes consisting of pyrimidinyl and phenyl rings, covalently bound to two electrodes.

## Results

**Figure 1:**
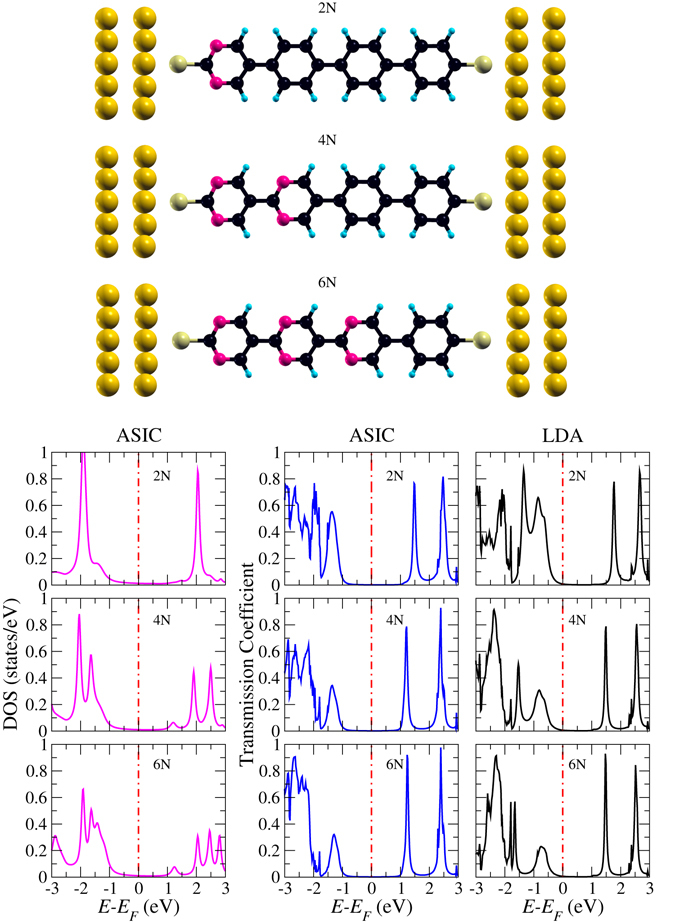
Atomic structures of the pyrimidinyl-triphenyl (2N), dipyrimidinyl-diphenyl (4N), and tripyrimidinyl-phenyl (6N) junctions. Blue, black, purple, yellow, and gold color denotes H, C, N, S, and Au atoms, respectively. Corresponding ASIC densities of states and zero-bias transmission are shown below. LDA results are given for comparison.

Figure 1 shows junctions with varying donor-acceptor ratio: pyrimidinyl-triphenyl, dipyrimidinyl-diphenyl, and tripyrimidinyl-phenyl. With reference to the number of involved N atoms (two in every pyrimidinyl ring), these configurations in the following will be called 2N, 4N, and 6N, respectively. Thiol groups are used to bind the molecules to the Au(111) electrodes, for which only the first two out of five atomic layers are shown in Fig. 1. We find that the most favorable binding site is the Au(111) hollow site, due to *sp* hybridization, which agrees with previous ab-initio calculations^21^. Moreover, we note that the planar structures of the molecules do not develop significant distortions in the geometry optimizations.

We first study the transport properties of the 2N, 4N, and 6N configurations by analyzing the zero-bias transmission coefficients as function of the energy, see Fig. 1. Both for the atomic self-interaction correction (ASIC) and local density approximation (LDA), the first transmission peak below the Fermi energy is significantly reduced when the number of pyrimidinyl rings increases (and thus the number of phenyl rings decreases), whereas all other peaks show only minor modifications. This observation can be explained by the fact that the amount of charge transfer from the donor to the acceptor region is reduced. Importantly, we find for the LDA transmission through the tails of the HOMO peaks at the Fermi energy, whereas the ASIC shifts those to lower energy. Since the ASIC provides qualitatively correct results, we will only consider those in the following. The fact that the difference between the ionization potential and electron affinity of the molecule in gas phase (4.43 eV) is higher than in the presence of the electrodes shows that the alignment of the molecular levels is substantially affected. According to the densities of states shown in Fig. 1, both the HOMO and HOMO–1 are dominated by N states (pyrimidinyl acts as acceptor). When the size of the pyrimidinyl region varies from 2N to 6N the HOMO splits into several peaks, because the N atoms in different distances to the electrode experience different chemical environments. The same mechanism also affects the LUMO, such that every peak belongs to the states created by additional N atoms.

**Figure 2:**
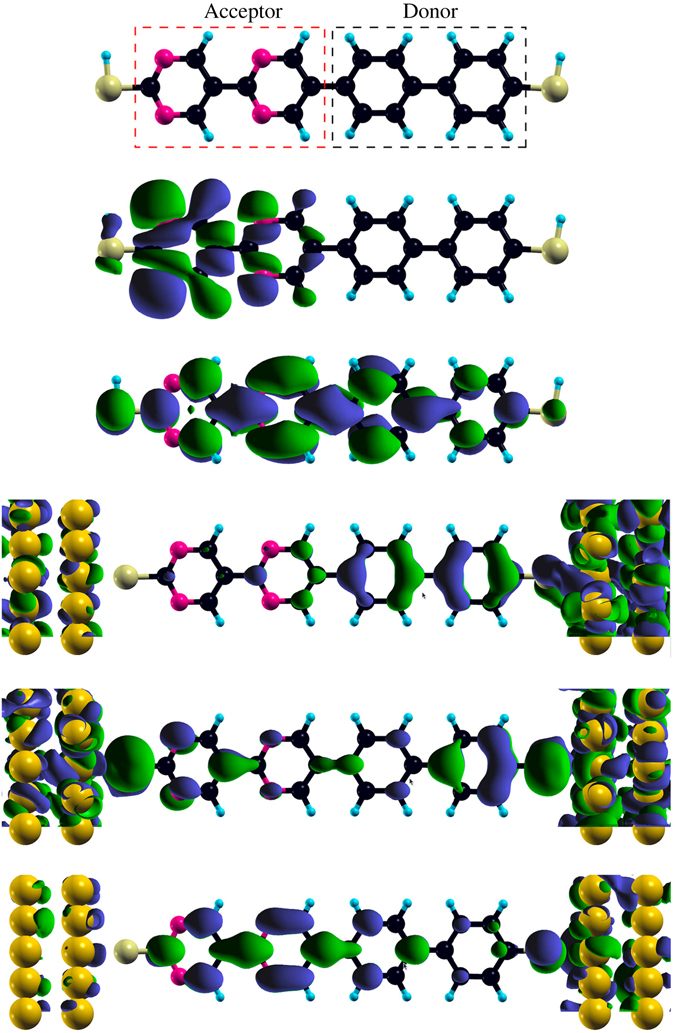
Dipyrimidinyl-diphenyl diode. Top to bottom: Acceptor and donor regions, HOMO, LUMO, transmission eigenchannels (obtained by diagonalizing the transmission matrix) of the HOMO–1, HOMO, and LUMO.

Figure 2 shows for the isolated dipyrimidinyl-diphenyl molecule (top) isosurface plots of the HOMO (2nd from top) and LUMO (3rd from top). The HOMO is localized in the acceptor region, whereas the LUMO is centered at the first pyrimidinyl ring after the acceptor-donor contact and extends over the whole molecule. As they are key for the transport, Fig. 2 shows in the lower part the HOMO–1, HOMO, and LUMO transmission eigenchannels (transmission from left to right). We observe in each case that the transmission relies mainly on the *π*-orbitals. The HOMO–1 transmission eigenchannel is localized on the phenyl region and the HOMO transmission eigenchannel on both ends of the molecule. On the other hand, the LUMO transmission eigenchannel is centered on the first pyrimidinyl ring after the acceptor-donor contact, similar to the LUMO itself.

**Figure 3:**
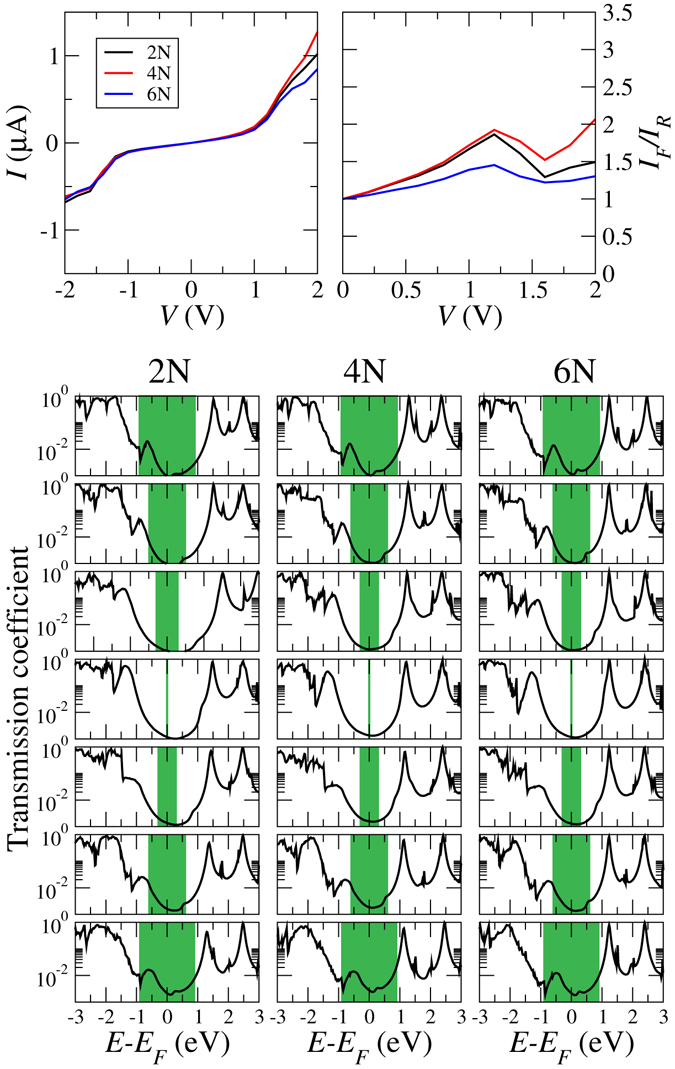
*I*-*V* characteristics, rectification behavior, and finite-bias (top to bottom: −1.8, −1.2, −0.6, 0, 0.6, 1.2, and 1.8 V) transmission coefficients of the 2N, 4N, and 6N configurations. The bias window is marked by green color.

We now turn to finite bias transport, see Fig. 3. We observe an asymmetry between positive and negative bias in the *I*-*V* characteristics, similar for all three configurations. Comparison of our results for the 4N configuration to ref. 9 shows excellent agreement with the experimental *I*-*V* characteristics, which demonstrates the predictive power of the employed computational approach. Figure 3 indicates that all the systems can be used for rectification, in each case with a distinct voltage dependence of the rectification ratio *I*_*F*_/*I*_*F*_, where *I*_*F*_ and *I*_*R*_ are the forward and reverse currents at the same voltage. The 4N configuration performs generally better than the 2N and 6N configurations as a consequence of enhanced forward current, which reflects different potential profiles across the molecular junctions due to different screening in the pyrimidinyl and phenyl rings.

The bias-dependent transmission coefficients displayed in Fig. 3 reveal under both positive and negative bias a clear shift of the HOMO peak towards the Fermi energy, accompanied by a loss of intensity. This corresponds to a high slope in the *I*-*V* characteristics when the HOMO peak enters the bias window. Moreover, since the LUMO peak shows hardly any shift, the HOMO-LUMO gap is reduced. The transport is hole dominated at low bias, because the tail of the HOMO peak contributes at the Fermi energy much more than that of the LUMO peak, which starts playing a role only at higher bias, see Fig. 3. This fact confirms the conjecture of ref. 9 that the transport through the 4N configuration must be due to holes. The transmission coefficients also allow us to understand the asymmetry of the *I*-*V* characteristics, as the overlap with the green bias window is larger for positive than for negative bias of the same magnitude. Finally, our data fit well to the argumentation in ref. 9 that the hole wavefunction behaves non-symmetrically under applied bias.

**Figure 4:**
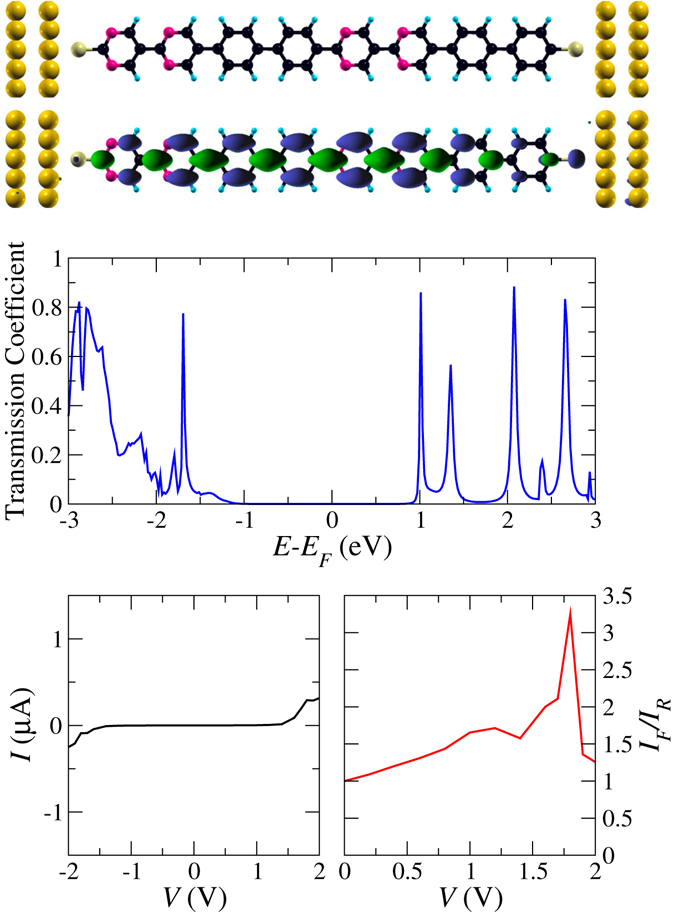
Atomic structure, LUMO transmission eigenchannel, zero-bias transmission, *I*-*V* characteristics, and rectification behavior of the tandem configuration.

Starting from the 4N configuration, which is so far the best performing system, we aim at impoving the rectification behavior. Since a serial connection of diodes appears to be a promising route, we couple two dipyrimidinyl-diphenyl molecules by means of a *σ*-type C-C bond in the transport direction, see the tandem configuration shown in Fig. 4. The zero-bias transmission coefficient reveals very close similarity to the 4N configuration concerning the HOMO-LUMO gap, see Fig. 1. The intensity of the HOMO peak is strongly reduced because of the serial arrangement of two molecules. Moreover, the transmission eigenchannel of the LUMO (at zero bias) is delocalized essentially over the whole molecule and declines towards the electrodes, which would not be expected from Fig. 2. Therefore, the system cannot be understood in terms of two coupled molecules but acts as an entity, similar to observations in refs 5, 22. As compared to the 4 N configuration, we obtain a higher threshold voltage of about 1.4 V, see Fig. 4, because the tail of the HOMO transmission peak is strongly suppressed (obvious on a logarithmic scale), and much better rectification at bias around 1.8 V.

In conclusion, we have investigated the dipyrimidinyl-diphenyl molecule and its derivatives in rectification applications. In particular, we have dealt with the roles of the acceptor and donor rings and the consequences when their ratio is changed. Serial connection of two molecules has been shown to improve the rectification behavior and threshold voltage as compared to a single molecule configuration. Rational design of molecular junctions by means of quantum transport simulations turns out to be a viable approach.

## Methods

We employ the Siesta^23^ implementation of density functional theory, which uses an atomic orbital basis set (double-zeta polarized basis for C, N, S, and Au with 30 meV global energy shift^24^). All core electrons are described by normconserving pseudopotentials of Troullier-Martins type^25^, including scalar relativistic corrections^26^, and a cutoff energy of 600 Ry is used. The ASIC is employed for the exchange-correlation functional in order not to underestimate the electron localization. Results obtained within the LDA^27^ are given for comparison only. Generally, the ASIC corrects the occupied states by downshifting them in energy and improves the level alignment between molecule and electrode as well as the HOMO-LUMO gap^10^. The parameter controlling the amount of ASIC added to the LDA (which is given by the screening provided by the chemical potential) is set to *α* = 0.7, a value that we have tested for the systems under consideration and that is known to give reliable results for related molecular systems^28^.

All structures are geometrically optimized with a force threshold of 0.02 eV/Å. Using the optimized geometries, we perform quantum electron transport calculations in the non-equilibrium Green’s function framework as implemented in the Smeagol package^29^, which builds up on Siesta and for which we use the same convergence criteria as for the electronic structure calculations. In the transport calculations the complex part of the integral yielding the charge density is computed using 16 energy points on the complex semi-circle, 16 points along the real axis, and 16 poles. For finite bias the integral over the real energies is evaluated for at least 500 points. An electronic temperature of 300 K is used in all calculations.

## References

[1] Tao NJ (2006). Electron Transport in Molecular Junctions. Nat. Nanotechnol..

[2] Darancet P, Widawsky JR, Choi HJ, Venkataraman L, Neaton JB (2012). Quantitative Current-Voltage Characteristics in Molecular Junctions from First Principles. Nano Lett..

[3] Aradhya SV, Venkataraman L (2013). Single-Molecule Junctions Beyond Electronic Transport. Nat. Nanotechnol..

[4] Perrin ML (2014). Large Negative Differential Conductance in Single-Molecule Break Junctions. Nat. Nanotechnol..

[5] Dell EJ, Capozzi B, Xia J, Venkataraman L, Campos LM (2015). Molecular Length Dictates the Nature of Charge Carriers in Single-Molecule Junctions of Oxidized Oligothiophenes. Nat. Chem..

[6] Aviram A, Ratner MA (1974). Molecular Rectifiers. Chem. Phys. Lett..

[7] Ng MK, Lee DC, Yu L (2002). Molecular Diodes Based on Conjugated Diblock Co-Oligomers. J. Am. Chem. Soc..

[8] Morales GM (2005). Inversion of the Rectifying Effect in Diblock Molecular Diodes by Protonation. J. Am. Chem. Soc..

[9] Díez-Pérez I (2009). Rectification and Stability of a Single Molecular Diode with Controlled Orientation. Nat. Chem..

[10] Obodo JT, Gkionis K, Rungger I, Sanvito S, Schwingenschlögl U (2013). Hydrogen Bonding as the Origin of the Switching Behavior in Dithiolated Phenylene-Vinylene Oligomers. Phys. Rev. B.

[11] Ding W, Negre CFA, Vogt L, Batista VS (2014). Single Molecule Rectification Induced by the Asymmetry of a Single Frontier Orbital. J. Chem. Theory Comput..

[12] Ding W (2015). Computational Design of Intrinsic Molecular Rectifiers Based on Asymmetric Functionalization of N-Phenylbenzamide. J. Chem. Theory Comput..

[13] Lörtscher E (2012). Transport Properties of a Single-Molecule Diode. ACS Nano.

[14] Chabinyc ML (2002). Molecular Rectification in a Metal-Insulator-Metal Junction Based on Self-Assembled Monolayers. J. Am. Chem. Soc..

[15] Danilov AV, Kubatkin SE, Kafanov SG, Flensberg K, Bjørnholm T (2006). Electron Transfer Dynamics of Bistable Single-Molecule Junctions. Nano Lett..

[16] Capozzi B (2015). Single-Molecule Diodes with High Rectification Ratios Through Environmental Control. Nat. Nanotechnol..

[17] Kotiuga M, Darancet P, Arroyo CR, Venkataraman L, Neaton JB (2015). Adsorption-Induced Solvent-Based Electrostatic Gating of Charge Transport Through Molecular Junctions. Nano Lett..

[18] Van Dyck C, Ratner MA (2015). Molecular Rectifiers: A New Design Based on Asymmetric Anchoring Moieties. Nano Lett..

[19] Nakamura H, Asai Y, Hihath J, Bruot C, Tao N (2011). Switch of Conducting Orbital by Bias-Induced Electronic Contact Asymmetry in a Bipyrimidinyl-Biphenyl Diblock Molecule: Mechanism to Achieve a pn Directional Molecular Diode. J. Phys. Chem. C.

[20] Li JC, Gong X (2013). Diode Rectification and Negative Differential Resistance of Dipyrimidinyl-Diphenyl Molecular Junctions. Org. Electron..

[21] Sellers H, Ulman A, Shnidman Y, Eilers JE (1993). Structure and Binding of Alkanethiolates on Gold and Silver Surfaces: Implications for Self-Assembled Monolayers. J. Am. Chem. Soc..

[22] Liu H (2011). Theoretical Investigation Into Molecular Diodes Integrated in Series Using the Non-Equilibrium Green’s Function Method. Phys. Chem. Chem. Phys..

[23] Junquera J, Paz Ó, Sánchez-Portal D, Artacho E (2001). Numerical Atomic Orbitals for Linear-Scaling Calculations. Phys. Rev. B.

[24] Soler JM (2002). The SIESTA Method for Ab Initio Order-N Materials Simulation. J. Phys. Condens. Matter..

[25] Troullier N, Martins JL (1991). Efficient Pseudopotentials for Plane-Wave Calculations. II. Operators for Fast Iterative Diagonalization. Phys. Rev. B.

[26] Kleinman L, Bylander DM (1982). Efficacious Form for Model Pseudopotentials. Phys. Rev. Lett..

[27] Ceperley DM, Alder BJ (1980). Ground State of the Electron Gas by a Stochastic Method. Phys. Rev. Lett..

[28] Filippetti A (2011). Variational Pseudo-Self-Interaction-Corrected Density Functional Approach to the Ab Initio Description of Correlated Solids and Molecules. Phys. Rev. B.

[29] Rocha AR (2005). Towards Molecular Spintronics. Nat. Mater..

